# Complete Genome Sequence of *Streptococcus suis* Serotype 16 Strain TL13

**DOI:** 10.1128/genomeA.00394-13

**Published:** 2013-06-27

**Authors:** Kaicheng Wang, Huochun Yao, Chengping Lu, Jiming Chen

**Affiliations:** China Animal Health and Epidemiology Center, Qingdao, Chinaa; College of Veterinary Medicine, Nanjing Agricultural University, Nanjing, Chinab

## Abstract

We report here the first complete genome sequence of *Streptococcus suis* serotype 16, which has been identified to be zoonotic. The sequenced strain TL13 was isolated from a pig in China. The genome is 2,038,146 bp in length, covering 1,950 coding sequences, 53 tRNAs, and 4 rRNA loci.

## GENOME ANNOUNCEMENT

*Streptococcus suis* is a peanut-shaped, Gram-positive bacterium and an important pig pathogen circulating in nearly all countries with an extensive pig industry. *S. suis* can be transmitted to humans through close contact with infected pigs. *S. suis* causes meningitis, septicemia, endocarditis, arthritis, and septic shock in pigs and humans. Human infection with *S. suis* has been reported in many European and Asian countries (1).

Based on variation in the bacterial capsular antigens, 33 serotypes of *S. suis*, namely serotypes 1 to 31, 33, and 1/2, have been identified (1). Among them, serotypes 1, 2, 4, 14, and 16 have caused fatal human infections (2–5). To date, genome sequences of *S. suis* have been reported only for serotypes 1, 2, 3, 7, 9, 14, and 1/2 (6). To facilitate studies and detection of *S. suis*, we sequenced the genome of an *S. suis* serotype 16 strain. The strain, TL13, was isolated from a clinically healthy pig in China and confirmed by a serotyping technique performed using serotyping antiserum prepared according to reported methods (7).

The complete genome sequence was determined by Solexa pyrosequencing at BGI (Shenzhen, China). Assembly was performed using SOAPdenovo. Gaps were filled by primer walking and sequencing of PCR products. The assembly of the genome was further verified by PCR. Coding sequences (CDS) were predicted using Glimmer 3.02 and GeneMarkS (8) and further examined with the nonredundant protein database through BLASTp. tRNAs and rRNAs were identified using tRNAscan-SE and RNAmmer (9), respectively.

The genome of strain TL13 consists of a single circular chromosome which is 2,038,146 bp in length, with a GC content of 41.32%. There are 1,950 CDS that account for 97.2% of the genome, 53 tRNAs, and 4 rRNA loci. The genome of TL13 harbors some virulence-associated genes, including *epf*, *pgdA*, *fbpS*, and *srtA* (10). There are 9 phage-related genes and 5 prophage elements in the TL13 genome.

The genome of *S. suis* TL13 was found to be in an orientation similar to that of the majority of the published genomes of *S. suis* in GenBank (6). The 89K pathogenicity island region in *S. suis* serotype 2 (11) was not found in the genome of TL13, but the 15-bp sequence for specific recombination event (12) was found in the TL13 genome. This suggested that TL13 has the potential to act as a recipient strain for the pathogenic island from the epidemic strain (6). In addition, two other genes, which produce muramidase-released protein and suilysin (13), were also absent in the genome of TL13.

### Nucleotide sequence accession number. 

The complete genome sequence of *S. suis* serotype 16 (strain TL13) has been assigned GenBank accession number CP003993.
